# Large-scale seroprevalence analysis of human metapneumovirus and human respiratory syncytial virus infections in Beijing, China

**DOI:** 10.1186/1743-422X-8-62

**Published:** 2011-02-10

**Authors:** Guilan Lu, Richard Gonzalez, Li Guo, Chao Wu, Jiang Wu, Guy Vernet, Gláucia Paranhos-Baccalà, Jianwei Wang, Tao Hung

**Affiliations:** 1 State Key Laboratory of Molecular Virology and Genetic Engineering, Institute of Pathogen Biology (IPB), Peking Union Medical College (PUMC) & Chinese Academy of Medical Sciences (CAMS), Beijing 100730, China; 2 Christophe Mérieux Laboratory, IPB, CAMS-Fondation Mérieux, Institute of Pathogen Biology, Peking Union Medical College & Chinese Academy of Medical Sciences, Beijing 100730, China; 3 Fondation Mérieux, Lyon 69002, France; 4 Beijing Centre for Disease Control and Prevention, Beijing 100013, China

## Abstract

**Background:**

Human metapneumovirus (hMPV), a recently identified virus, causes acute respiratory tract infections (ARTIs) in infants and children. However, studies on the seroepidemeology of hMPV are very limited in China. To assess the seroprevalence of hMPV infection in China, we tested a total of 1,156 serum specimens for the presence of anti-hMPV IgG antibody in children and adults free of acute respiratory illness in Beijing, China by using hMPV nucleocapsid (N) protein as an antigen. As a control, we used the human serum antibody against the N protein of human respiratory syncytial virus (hRSV), the most important viral agent responsible for ARIs in children.

**Results:**

The seropositive rate for hMPV increased steadily with age from 67% at 1-6 mo to 100% at age 20. However, the rate dropped slightly between 6 mo and 1 yr of age. The seropositive rate for hRSV also increased steadily with age from 71% at 1-6 mo to 100% at age 20. In children aged six months to six years, the seropositive rates for the anti-hRSV IgG antibody were significantly higher than those for hMPV. Additionally, IgG antibody titers to hMPV and hRSV were significantly higher in adults than in young children. Consistent with the seropositive rates, the geometric mean titer of anti-hMPV IgG antibody was lower than that of anti-hRSV IgG antibody in children aged six months to six years.

**Conclusions:**

Our results indicate that similar to hRSV, exposure to hMPV is ubiquitous in the Beijing population. However, the seroprevalence of anti-hMPV IgG antibody is lower than that of hRSV in children between six months and six years old, which suggests a different number of repeat infections or a different response to infections.

## Background

Human metapneumovirus (hMPV), thought to belong to the *Metapneumovirus *genus of the *Pneumovirinae *subfamily, is a recently identified human respiratory pathogen first isolated from hospitalized children with acute respiratory infections (ARIs) in the Netherlands [1]. The viral genome, clinical manifestations, and epidemiology associated with hMPV are similar to those of human respiratory syncytial virus (hRSV), which was identified in 1956 and is the most important viral agent responsible for ARIs in children [2-4].

Since its initial identification, hMPV infections have been reported worldwide. However, fluctuating incidence of its infection has been reported by groups from different areas, varying from 2.2% to 43% in respiratory tract samples from patients with ARIs [5-16]. Seroepidemiological investigations also showed that the prevalence of hMPV may differ between geographical locations. For example, in the Netherlands, virtually all children have been exposed to hMPV by the age of five, demonstrating that hMPV infection is common in childhood [1]. In Canada, an ELISA-based test using recombinant nucleocapsid protein (N protein) of hMPV produced by baculovirus revealed that more than 90% of patients over 16 years of age tested seropositive for hMPV [17]. In China, however, studies on the seroepidemeology of hMPV have been limited, and it is unclear what percentage of the population in different age groups have been infected with the virus.

The N protein, about 394 amino acids in length, is encoded by the N gene of hMPV genome. The hMPV N protein is abundantly expressed during the early replication stage of the virus and can stimulate a sustained immune response [18]. Because the amino acid identity of hMPV N is highly conserved within and between the A and B subgroups of hMPV [19], it has been widely applied in the immunoassay of hMPV infection and in the investigation of seroprevalence of hMPV infections [17,18]. hMPV N protein shares 42-44% homology of amino acid sequence with hRSV [19]. As previous studies have not shown obvious cross-reactions in immunoassays such as ELISA, the hRSV N protein has been used as a reference to evaluate the seroprevalence of hMPV [18,20].

To assess the seroprevalence of hMPV infection in China, we used hMPV N protein as an antigen to test serum samples for the presence of anti-hMPV IgG antibody in children and adults free of acute respiratory illness in Beijing, China. The IgG antibody against N protein of hRSV was tested in parallel as a control. Our results indicate that exposure to hMPV is ubiquitous in the Beijing population. Lower seropositive rates and geometric mean titer (GMT) of anti-hMPV IgG were observed in children aged six months to six years when compared to hRSV. This may reflect the divergence of infection pattern between hMPV and hRSV in children. Our data will inform the evaluation of the social and economic burden of hMPV infection and enable the development of medical or public health strategies to combat hMPV infection in the population.

## Results

### Establishment of an ELISA-based detection method for seroprevalence of hMPV and hRSV IgG antibody

**Figure 1:**
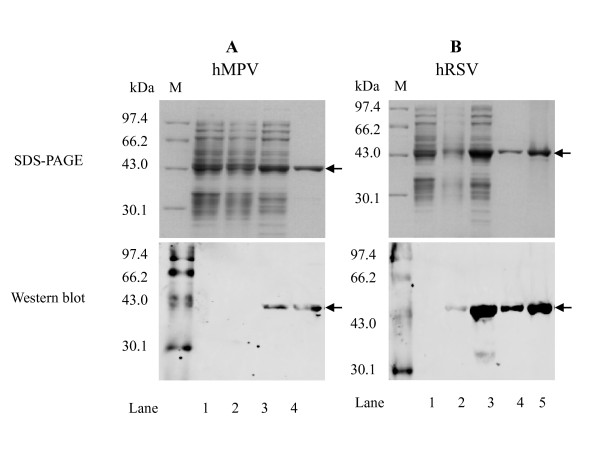
**Expression and purification of hMPV and hRSV N proteins**. His-tagged N proteins from *E. coli *BL21 (DE3) cell lysates or purified N proteins were examined by SDS-PAGE (upper panels) and Western blot analysis (bottom panels). Arrows indicate N protein bands and the bands detected by anti-His monoclonal antibody (Panel A, lane 3, 4; Panel B, lane 3, 4, 5). (A) Expression and purification of hMPV N protein. Lane 1, total protein extracted from *E. coli *BL21 (DE3) transformed with pET30a (+); lane 2, supernatant fraction from *E. coli *BL21 (DE3) transformed with pET-30a(+)-hMPV-N without IPTG induction; lane 3, supernatant fraction from *E. coli *BL21 (DE3) transformed with pET-30a(+)-hMPV-N induced by IPTG; lane 4, purified 6×His-tagged hMPV-N protein. (B) Expression and purification of hRSV N protein. Lane 1, total protein extracted from *E. coli *BL21 (DE3) transformed with pET30a (+); lane 2, supernatant fraction from *E. coli *BL21 (DE3) transformed with pET-30a(+)-hRSV-N without IPTG induction; lane 3, supernatant fraction from *E. coli *BL21 (DE3) transformed with pET-30a(+)-hRSV-N induced by IPTG; lane 4, hRSV-N protein purified using HisTrap Q FF column; lane 5, hRSV-N protein purified using HisTrap HP column. Lane M, molecular protein markers.

Both recombinant hMPV and hRSV N proteins were effectively expressed in *E. coli *BL21 (DE3) as soluble proteins. Recombinant hMPV N protein was then purified from BL21 (DE3) cell lysates by Ni-chelating chromatography, and recombinant hRSV N protein was purified by anion exchange chromatography followed by Ni-chelating chromatography (data not shown). The purity of both recombinant N proteins was greater than 90%. The purified proteins were confirmed by Western blot analysis using mouse anti-6×His tag monoclonal antibody as the primary antibody and IRDye 680 goat anti-mouse monoclonal antibody as the secondary antibody (Figure 1).

**Figure 2:**
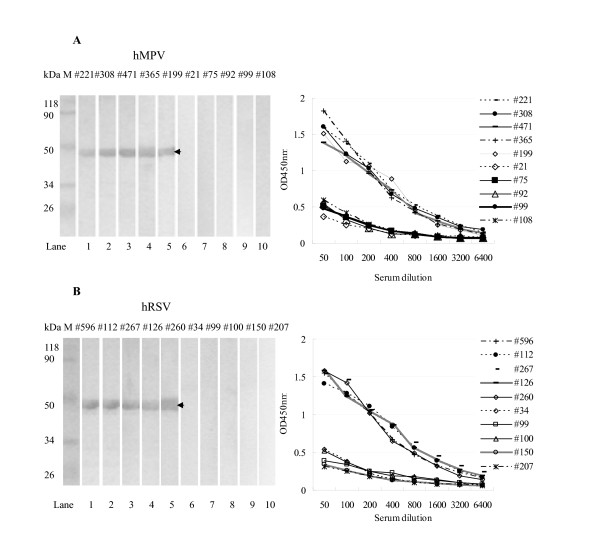
**Screening of hMPV or hRSV-specific IgG positive and negative human sera samples**. Examples of samples that are positive or negative for hMPV or hRSV. (A) Screening of hMPV positive and negative sera samples. Left, Western blot analysis was used to show the expression of hMPV N protein in positive sera (#221, #308, #471, #365, #199) and negative sera (#21, #75, #92, #99, #108). Right, two-fold serial dilutions of positive sera (#221, #308, #471, #365, #199) and negative sera (#21, #75, #92, #99, #108) were tested in plates coated with purified hMPV N protein as described in *Methods *section. (B) Screening of hRSV positive and hRSV negative sera samples. Left, Western blot analysis was used to show the expression of hRSV N protein in positive sera (#596, #112, #267, #126, #260) and negative sera (#34, #99, #100, #150, #207). Right, two-fold serial dilutions of positive sera (#596, #112, #267, #126, #260) and negative sera (#34, #99, #100, #150, #207) were tested in plates coated with purified hRSV N protein as described in *Methods *section. Arrows indicate positive bands.

To optimize ELISA conditions, we performed chessboard titration tests using the recombinant hMPV and hRSV N proteins. hMPV-positive and negative sera samples were identified by Western blot analysis (Figure 2A, left panel). The optimal antigen (hMPV N protein) concentration for ELISA under our test conditions was determined to be 0.25 μg/ml for hMPV, because at a 1:200 serum dilution, the OD_450 nm _value of positive sera was approximately 1.0 and the difference between the OD_450 nm _value of positive and negative sera was greatest (Figure 2A, right panel). Similarly, we determined the optimal assay conditions for hRSV N protein-ELISA to be 0.125 μg/ml hRSV N protein at 1:200 serum dilution (Figure 2B).

Based on these ELISA conditions, we then determined the cut-off value for these assays. Twenty-eight anti-hMPV IgG negative and 20 anti-hRSV IgG negative sera samples were identified using Western blot analysis of 100 sera samples randomly collected from children under five years of age. The OD_450 nm _of each negative sample was determined by ELISA for hMPV N protein or hRSV N protein. The mean value of the OD_450 nm _of the hMPV negative sera was 0.24 with a standard deviation (SD) of 0.033. The mean value of the OD_450nm _of the hRSV negative sera was 0.261 with a SD of 0.034. Based on the formula: cut-off value = mean OD450 nm of the negative sera + three fold SDs [21,22], the hMPV and hRSV positive cut-off values were then defined as 0.339 and 0.363. A tested sample was scored as "positive" if its OD450 nm value was above the cut-off value.

The sensitivity of these two ELISA methods was determined using purified murine IgG against hMPV or hRSV N proteins. The limits of detection were 0.0625 μg/ml IgG and 0.125 μg/ml IgG, respectively.

To test the specificity of the ELISA methods established in this study, the reactions of mouse sera against influenza virus A (subtypes H1-H16), human coronaviruses (229E, HKU1 and NL63), and polyomavirus JC against hMPV and hRSV N protein were evaluated. There was no obvious cross-reaction between those mouse antibodies and the hMPV or hRSV N proteins (data not shown). Furthermore, we did not observe cross-reaction between the hRSV N protein and hMPV N protein (data not shown).

### Seropositive rates of hMPV and hRSV

**Figure 3:**
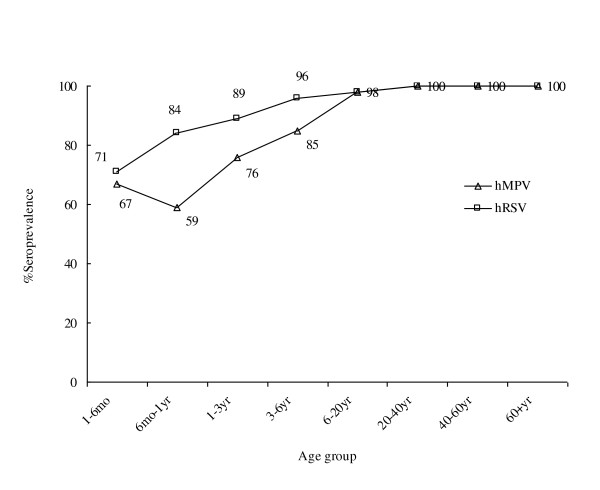
**Seroprevalence of IgG antibodies against hMPV and hRSV**. A total of 1,156 serum specimens were tested. Anti-hMPV and anti-hRSV IgG antibodies were detected using the N-ELISA method (see Methods) at a dilution of 1:200. All serum samples were grouped based on age, as indicated at the bottom of each panel. The percentage of positive samples in each group is listed above each data point.

For large-scale ELISA screening, each of the tested serum samples was evaluated for the presence of anti-hMPV and anti-hRSV (N protein) antibodies. Our analysis indicates that among the 1,156 samples, 67% (28/42) of subjects aged 1-6 mo, 59% (34/58) of subjects aged 6 mo-1 yr, 76% (65/85) aged 1-3 yr, 85% (79/93) aged 3-6 yr, 98% (119/122) aged 6-20 yr, and 100% (756/756) aged 20-80 yr were seropositive for hMPV; whereas 71% (30/42) of subjects aged 1-6 mo, 84% (49/58) aged 6 mo-1 yr, 89% (76/85) aged 1-3 yr, 96% (89/93) aged 3-6 yr, 98% (119/122) aged 6-20 yr, and 100% (756/756) aged 20-80 yr were seropositive for hRSV (Figure 3 and Table 1). Comparison between the hMPV and hRSV-positive sera by statistical analysis (χ^2 ^tests) demonstrated that the seropositive rates of hMPV were significantly lower than those of hRSV in the age groups of six months to six years, whereas no significant difference was observed in the age groups of one to six months, or six years to 80 years (Table 1).

**Table 1:** Age Seropositivity Profiles of hMPV and hRSV

	hMPV	hRSV
Age	% positive (no. positive/no. tested)	% positive (no. positive/no. tested)
1-6 mo	67 (28/42)	71(30/42)
6 mo-1 yr	59 (34/58)*	84 (49/58) *
1-3 yr	76 (65/85)*	89 (76/85) *
3-6 yr	85 (79/93)*	96 (89/93) *
6-20 yr	98 (119/122)	98 (119/122)
20-40 yr	100 (248/248)	100 (248/248)
40-60 yr	100 (328/328)	100 (328/328)
60+ yr	100 (180/180)	100 (180/180)

* *P *< 0.05, by χ^2 ^test.

### Titer of anti-hMPV and anti-hRSV IgG antibodies

**Figure 4:**
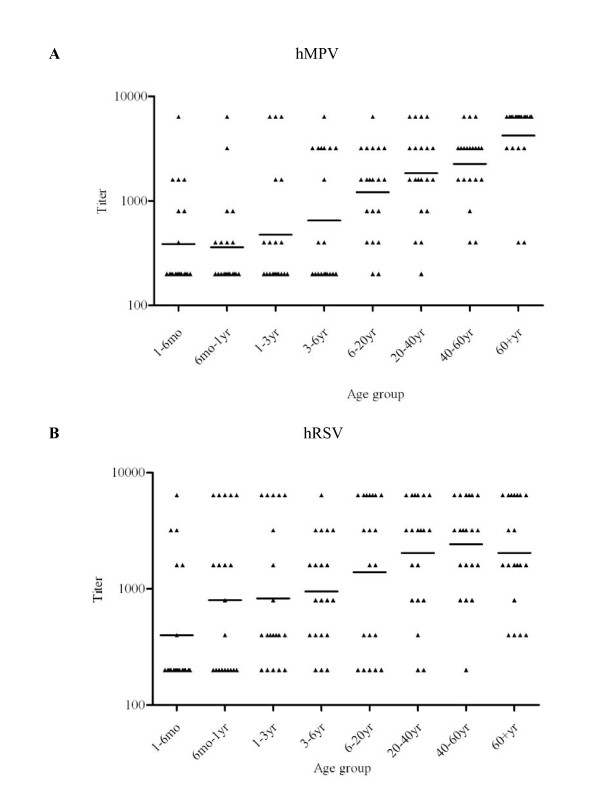
**Titers of anti-hMPV and anti-hRSV IgG antibodies**. Twenty human serum samples that were seropositive for hMPV (A) or hRSV (B) were randomly selected from each age group, for a total of 160 samples. ELISA was performed using hMPV N protein or hRSV N protein as the antigen. Titers are shown for the indicated age groups. Horizontal bars indicate the GMTs of antibodies against hMPV or hRSV.

**Figure 5:**
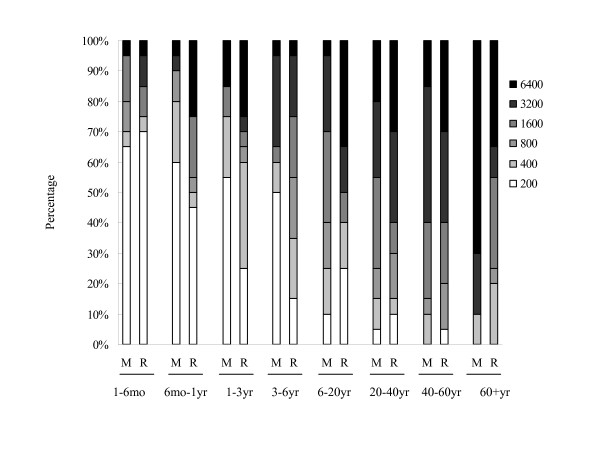
**Proportions of tested serum samples with different titers of anti-hMPV and anti-hRSV antibodies within different age groups**. Cumulative percentages of anti-hMPV and anti-hRSV antibodies detected in samples from each age group. The percentage of samples with corresponding titers of anti-hMPV and anti-hRSV IgG antibodies are indicated by different colors defined in the figure key. Titers are expressed as the reciprocal of the fold dilution. A total of 160 human serum samples that were seropositive for hMPV or hRSV were randomly selected for ELISA using hMPV N protein or hRSV N protein as the antigen. M, hMPV; R, hRSV.

To characterize the seroprevalence of hMPV infection in the sample used in this study further, we analyzed the titers of anti-hMPV and anti-hRSV IgG antibodies in our samples. Twenty randomly selected seropositive samples in each age group were analyzed. The geometric mean titers (GMTs) of anti-hMPV IgG and anti-hRSV IgG were significantly higher in the age group of >20 years than in the age group of ≤20 years (for GMT of anti-hMPV IgG, 556 vs 2599, *P *= 0.000; for anti-hRSV IgG, 811 vs 2160, *P *= 0.000, Mann-Whitney U test) (Figure 4). Consistent with the seropositive rates, the GMT of anti-hMPV IgG (481) was also lower than that of anti-hRSV IgG (857) in a younger group between six months and six years of age (Mann-Whitney U test, *P *= 0.005). In this age group, the proportion of specimens with a low anti-hMPV IgG titer (≤1:400) was significantly higher than those with a low anti-hRSV IgG titer (71.6% vs 48.3%, χ^2 ^test, *P *= 0.009) (Figure 5). Notably, for individuals over six years of age, the proportion of specimens with a high anti-hMPV titer (≥1:3200) increased and was comparable to those with a high anti-hRSV IgG titer (56.25% vs 53.75%, χ^2 ^test, *P *= 0.751) (Figures 4 and 5).

## Discussion

In this report, we show a high seroprevalence of anti-hMPV IgG antibody in the Beijing area. The 1,156 serum samples included in our study were randomly collected in 2008, from individuals receiving routine physical examinations and who were free of recent respiratory infections. Thus, our data should represent the incidence of hMPV infection in Beijing. Our results indicate that virtually all children over the age of six years have been exposed to hMPV infection. Compared to reports from the Netherlands and Japan in which 192 and 142 samples were tested [1,23], our data were derived from a larger number of subjects. Consistent with those two reports, our results suggest that hMPV infection is ubiquitous. However, our results showed a higher rate of hMPV infection in infants and younger children than reported in the Netherlands or Japan. We found that the seroprevalence of hMPV in children aged 6 mo to 1 yr was 59% compared to 25% in the Netherlands and 17.7% in Japan. Thus, our data suggests that primary infection of children by hMPV may occur much earlier in Beijing than in the Netherlands or Japan. This discrepancy may be caused by differences in exposure to hMPV in different geographical regions, or by other factors such as social, environmental, or climate conditions.

Similarly, hRSV has a high seroprevalence rate in Beijing. Our data correlate with previous findings that seroprevalence increases with age and that by the age of three, over 90% of children are seropositive for hRSV [24]. Interestingly, our results demonstrate that the seropositive rate of hMPV in children six months to six years of age are significantly lower than seropositive rates of hRSV (P < 0.05), suggesting that hRSV infection occurs earlier than hMPV infection.

Our results showed that the seropositivity of hMPV decreases during the 6 mo-1 year period. Maternal antibodies may be responsible for higher hMPV seropositivity in individuals 1-6 months old. The seropositivity of hMPV decreases during the 6 mo-1 year period as the level of the maternal antibody decreases. The antibody titers then increase with age, after repeat hMPV infection.

Notably, within the age group of 6 mo to 6 yr, the GMTs of anti-hMPV IgG were significantly lower than those of anti-hRSV IgG. Significantly more specimens had a low anti-hMPV IgG (≤1:400) titer than had a low anti-hRSV IgG titer. The reasons for this disparity are unclear. It is possible that infection by hMPV occurs later than infection by hRSV, thus leading to the delayed increase in the seropositive rate and antibody titer of hMPV [25]. It is likely that less response to hMPV or more exposure to hRSV that would boost the antibody titer, results in a higher antibody titer against hRSV than against hMPV. However, large-scale investigations using samples collected from different geographic regions are necessary to distinguish the immunological responses to the two viruses during early human life. We did not find significant differences between the seroprevalence of hMPV and hRSV infections in children less than six months of age. This reflects high levels of maternal antibody against hMPV and hRSV, which may be attributed to the high prevalence of antibodies in adults (Table 1, Figure 3 and 4).

To determine the specificity of our ELISA method, we tested for reactions between hMPV or hRSV N protein and murine antibodies against multiple viruses, including influenza A viruses (H1-H16), human coronaviruses (229E, HKU1, NL63) and polyomavirus JC. We found no obvious reactivity in ELISA assays. Additionally, we did not detect a cross-reaction between hMPV N protein and hRSV N protein. These results indicate that the N protein-based ELISA specifically detects both anti-hMPV and anti-hRSV antibodies. However, we need to further evaluate the specificity of the N protein-based ELISA by using antibodies against other respiratory virus antigens. The sensitivity of the ELISA method for detecting hMPV and hRSV was determined by using purified murine specific anti-hMPV or hRSV N IgG, respectively. To our knowledge, limits of detection comparable to the sensitivity achieved by our methods have not been reported in other serological surveys of hMPV.

Repeated infections of an individual by hMPV and hRSV have been reported [26-29], suggesting that immunity against hMPV and hRSV is not solid, is transient, or is incomplete against heterologous strains. The high prevalence and high titers of hMPV and hRSV that we observed in adults may suggest that re-infection by hMPV and hRSV occurs throughout life [23,30].

## Conclusions

Our results suggest that similar to hRSV infection, hMPV infection is ubiquitous in the Beijing population. However, the seroprevalence of the IgG antibody against hMPV is lower than that against hRSV in children between six months and six years of age, which may reflect differences in infection pattern between the two viruses. Our findings provide further information to aid the development of strategies to control and prevent hMPV infection.

## Methods

### Expression and purification of recombinant hMPV and hRSV N proteins

Viral RNAs from a nasopharyngeal swab sample positive for hMPV (#1963) or RSV-B-N (#1949) were extracted. Full-length N genes were reverse-transcribed and cloned into pET30a (+) containing a 6×His tag downstream of a T7 promoter (Novagen, Wisconsin, USA). These recombinant constructs were transformed into *E. coli *BL21 (DE3) (Novagen), and the transformants were grown in LB medium containing 50 μg/ml kanamycin at 37°C for 16 hours. The cells were diluted to 1:100, transferred to fresh LB medium, and grown at 37°C until the OD600nm reached 0.8. To induce expression of recombinant proteins, cells were incubated with 1 mM IPTG at 16°C overnight. hMPV N protein-expressing cells were harvested and centrifuged at 2,500 × g for 15 minutes at 4°C, resuspended in lysis buffer (20 mM phosphate, 500 mM NaCl, 20 mM imidazole, pH7.4), and sonicated at 4°C. The lysates were centrifuged at 30,000 × g for 15 minutes at 4°C. The supernatant was purified using a HisTrap HP 1 ml column (GE Healthcare, Waukesha, USA). Cells expressing the hRSV N protein were harvested by centrifugation at 2,500 × g for 15 minutes at 4°C, resuspended in lysis buffer (20 mM phosphate at pH7.4), and then sonicated at 4°C. The lysates were centrifuged at 30,000 × g for 15 minutes at 4°C. The supernatant was purified using a HisTrap Q FF 5 ml column (GE Healthcare) followed by a HisTrap HP 1 ml column (GE Healthcare). The purification of N proteins was evaluated by 12% SDS-PAGE and optical density scanning using the Gene Genius Bio-imaging System (Syngene, Synoptics Ltd. Cambridge. UK). The yields of purified 6×His-tagged recombinant proteins were quantified using the Pierce^®^BCA Protein Assay Kit (Thermo Scientific, Rockford, USA).

### Serum samples

Serum specimens were collected from 1,156 subjects, including 400 children (from the Beijing Children's Hospital) and 756 adults (from the Beijing Blood Center), during routine physical examinations in 2008. All individuals were free of acute respiratory infection for at least three months prior to sampling. All samples were collected after obtaining informed consent either from the individuals or from the individual's guardians. The sera were separated immediately after collection, stored at -80°C, and inactivated at 56°C for 30 minutes before use.

### Western blot analysis

Purified recombinant proteins were separated by 12% SDS-PAGE and transferred onto a nitrocellulose membrane, as previously described [31]. The membrane was blocked for two hours in 5% non-fat milk. Mouse anti-His monoclonal antibody (Sigma, Munich, Germany) diluted 1:3000 in non-fat milk or serum samples diluted 1:50 in non-fat milk were added, and membranes were incubated for one hour at room temperature. Membranes were then washed three times with PBS-0.1% Tween and incubated for one hour at room temperature with the secondary antibody, IRDye 680 goat anti-mouse monoclonal antibody (Li-COR Biosciences, Nebraska, USA) or horseradish peroxidase-conjugated goat anti-human IgG monoclonal antibody (Sigma, Munich, Germany). Subsequently, membranes were washed three times with PBS-0.1% Tween, then developed using Odyssey (Li-COR Biosciences) or a tetramethylbenzidine (TMB) substrate (Thermo Scientific, Rockford, USA) according to the manufacturers' instructions.

### Enzyme-linked immunosorbent assay (ELISA)

Chessboard titration tests were conducted using positive and negative serum samples that were randomly selected. Western blot analysis was used to determine the optimal concentration of the coated antigen and serum dilution. Subsequently, 96-well plates (Costar, Bethesda, USA) were coated with 100 μl of 0.25 μg/ml purified hMPV N proteins or 0.125 μg/ml purified hRSV N proteins, in 0.05 M sodium hydrogen carbonate buffer (pH9.6). Plates were incubated overnight at 4°C, then blocked with 1% BSA overnight at 4°C and washed three times with PBS-0.3% Tween. Subsequently, 100 μl of a 1:200 dilution of serum specimens was added and incubated at 37°C for one hour. The plates were then washed six times with PBS-0.3% Tween and incubated for one hour at 37°C with horseradish peroxidase-conjugated goat anti-human IgG (Sigma) diluted 1:40,000. The plates were washed again six times with the same solution, and antibodies were detected by adding 100 μl substrate solutions A and B (Wantai Biotech Corp. Beijing, China) followed by incubation at 37°C for 10 minutes. The reactions were terminated by adding 50 μl of 2 M H_2_SO_4_. Optical densities (OD) were read at 450 nm (OD_450 nm_). The average OD values for the hMPV-negative human sera samples (n = 28), shown to be negative by Western blot analysis for the hMPV-N protein, were measured. The average OD values of the hRSV-negative human sera samples (n = 20), shown to be negative by Western blot analysis for the hRSV-N protein, were also measured. The cut-off values were defined as the mean OD of the negative sera plus three standard deviations [21,22]. Samples with OD_450 nm _values above the cut-off value were considered positive.

To determine the specificity of our ELISA method, we tested mouse sera against inflenza virus HA proteins (subtype H1-H16), human coronavirus (229E, HKU1, NL63) N proteins, and polyomavirus JC VP1 protein for reaction against hMPV and hRSV proteins. Samples of sera were tested in serial dilutions of two-fold, starting at a 1:1000 dilution.

To determine the sensitivity of ELISA method established in this study, mice sera against hMPV N protein and hRSV N protein were purified using protein-G sepharose column (GE Healthcare) and quantified using Pierce^®^BCA Protein Assay Kit (Thermo Scientific, Rockford). The purified murine IgG against hMPV or hRSV were tested in serial dilutions of two-fold (starting at as 1 μg/ml concentration).

In addition, 20 randomly selected positive serum samples in each group were subjected to anti-hMPV and anti-hRSV titer assays. To determine the endpoints of antibody titers, titers were calculated as the highest dilution of a serum showing an OD_450 nm _reading of two times the mean of the negative serum control (starting at a 1:50) [32].

### Statistical analysis

Seropositive rates were evaluated using χ2 tests. Mean antibody titers between children and adults positive for hMPV and hRSV exposure were analyzed using the Mann-Whitney U test. A *P *value ≤ 0.05 was considered statistically significant.

## Competing interests

The authors declare that they have no competing interests.

## Authors' contributions

GL, RG, and JW designed the study. GL, LG, and CW conducted the experiments. JW was in charge of the collection of sera specimens. GL, RG, GP, GV and JW wrote the manuscript, and GV, GP, JW and TH revised the manuscript. All authors have read and approved the final manuscript.

## References

[B1] Van den Hoogen BG de Jong JC Groen J Kuiken T de Groot R Fouchier RA Osterhaus AD A newly discovered human pneumovirus isolated from young children with respiratory tract disease Nat Med 2001 7 719 724 10.1038/89098 11385510 PMC7095854

[B2] Shay DK Holman RC Newman RD Liu LL Stout JW Anderson LJ Bronchiolitis-associated hospitalizations among US children, 1980-1996 JAMA 1999 282 1440 1446 10.1001/jama.282.15.1440 10535434

[B3] Esper F Martinello RA Boucher D Weibel C Ferguson D Landry ML Kahn JS A 1-year experience with human metapneumovirus in children aged <5 years J Infect Dis 2004 189 1388 1396 10.1086/382482 15073675 PMC7109939

[B4] Boivin G De Serres G Côté S Gilca R Abed Y Rochette L Bergeron MG Déry P Human metapneumovirus infections in hospitalized children Emerg Infect Dis 2003 9 634 640 12781001 10.3201/eid0906.030017 PMC3000156

[B5] Maggi F Pifferi M Vatteroni M Fornai C Tempestini E Anzilotti S Lanini L Andreoli E Ragazzo V Pistello M Specter S Bendinelli M Human metapneumovirus associated with respiratory tract infections in a 3 year study of nasal swabs from infants in Italy J Clin Microbiol 2003 41 2987 2991 10.1128/JCM.41.7.2987-2991.2003 12843031 PMC165353

[B6] García-García ML Calvo C Pérez-Breña P De Cea JM Acosta B Casas I Prevalence and clinical characteristics of human metapneumovirus infections in hospitalized infants in Spain Pediatr Pulmonol 2006 41 863 871 16850437 10.1002/ppul.20456 PMC7167809

[B7] Stockton J Stephenson I Fleming D Zambon M Human metapneumovirus as a cause of community-acquired respiratory illness Emerg Infect Dis 2002 8 897 901 12194763 10.3201/eid0809.020084 PMC2732534

[B8] Williams JV Harris PA Tollefson SJ Halburnt-Rush LL Pingsterhaus JM Edwards KM Wright PF Crowe JE Jr Human metapneumovirus and lower respiratory tract disease in otherwise healthy infants and children N Engl J Med 2004 350 443 450 10.1056/NEJMoa025472 14749452 PMC1831873

[B9] Mullins JA Erdman DD Weinberg GA Edwards K Hall CB Walker FJ Iwane M Anderson LJ Human metapneumovirus among children hospitalized for acute respiratory illness Emerg Infect Dis 2004 10 700 705 15200863 10.3201/eid1004.030555 PMC3323105

[B10] Peiris JS Tang WH Chan KH Khong PL Guan Y Lau YL Chiu SS Children with respiratory disease associated with metapneumovirus in Hong Kong Emerg Infect Dis 2003 9 628 633 12781000 10.3201/eid0906.030009 PMC3000155

[B11] Aberle JH Aberle SW Redlberger-Fritz M Sandhofer MJ Popow-Kraupp T Human metapneumovirus subgroup changes and seasonality during epidemics Pediatr Infect Dis J 2010 29 1016 1018 20489673 10.1097/INF.0b013e3181e3331a

[B12] Aberle SW Aberle JH Sandhofer MJ Pracher E Popow-Kraupp T Biennial spring activity of human metapneumovirus in Austria Pediatr Infect Dis J 2008 27 1065 1068 10.1097/INF.0b013e31817ef4fd 18978517

[B13] Ali SA Williams JV Chen Q Faori S Shehabi A Jundi EA Khuri-Bulos N Halasa N Human metapneumovirus in hospitalized children in Amman, Jordan J Med Virol 2010 82 1012 1016 10.1002/jmv.21768 20419816 PMC3347978

[B14] Kim CK Choi J Callaway Z Kim HB Chung JY Koh YY Shin BM Clinical and epidemiological comparison of human metapneumovirus and respiratory syncytial virus in seoul, Korea, 2003-2008 J Korean Med Sci 2010 25 342 347 10.3346/jkms.2010.25.3.342 20191030 PMC2826723

[B15] IJpma FF Beekhuis D Cotton MF Pieper CH Kimpen JL van den Hoogen BG van Doornum GJ Osterhaus DM Human metapneumovirus infection in hospital referred south Afirca children J Med Virol 2004 73 486 493 10.1002/jmv.20116 15170647

[B16] Williams JV Crowe JE Jr Enriquez R Minton P Peebles RS Jr Hamilton RG Higgins S Griffin M Hartert TV Human metapneumovirus infection plays an etiologic role in acute asthma exacerbations requiring hospitalization in adults J Infect Dis 2005 192 1149 1153 10.1086/444392 16136455 PMC1476781

[B17] Liu L Bastien N Sidaway F Chan E Li Y Seroprevalence of human metapneumovirus(hMPV) in the Canadian Province of Saskatchewan analyzed by a recombinant nucleocapsid protein-based enzyme-linked immunosorbent assay J Med Virol 2007 79 308 313 10.1002/jmv.20799 17245714

[B18] Hamelin ME Boivin G Development and validation of an enzyme-linked immunosorbent assay for human metapneumovirus serology based on a recombinant viral protein Clin Diagn Lab Immunol 2005 12 249 253 15699418 10.1128/CDLI.12.2.249-253.2005 PMC549303

[B19] Bastien N Normand S Taylor T Ward D Peret TC Boivin G Anderson LJ Li Y Sequence analysis of the N, P, M and F genes of Canadian human metapneumovirus strains Virus Res 2003 93 51 62 10.1016/S0168-1702(03)00065-0 12727342 PMC7172423

[B20] Okamoto M Sugawara K Takashita E Muraki Y Hongo S Mizuta K Itagaki T Nishimura H Matsuzaki Y Development and evaluation of a whole virus-based enzyme-linked immunosorbent assay for the detection of human metapneumovirus antibodies in human sera J Virol Methods 2010 164 24 29 10.1016/j.jviromet.2009.11.019 19925829

[B21] Kahn JS Kesebir D Cotmore SF D'Abramo A Jr Cosby C Weibel C Tattersall P Seroepidemiology of human bocavirus defined using recombinant virus-like particles J Infect Dis 2008 198 41 50 10.1086/588674 18491974

[B22] He Y Zhou Y Siddiqui P Niu J Jiang S Identification of immunodominant epitopes on the membrane protein of the severe acute respiratory syndrome-associated coronavirus J Clin Microbiol 2005 43 3718 3726 10.1128/JCM.43.8.3718-3726.2005 16081901 PMC1234014

[B23] Ebihara T Endo R Kikuta H Ishiguro N Yoshioka M Ma X Kobayashi K Seroprevalence of human metapneumovirus in Japan J Med Virol 2003 70 281 283 10.1002/jmv.10391 12696118

[B24] Cox MJ Azevedo RS Cane PA Massad E Medley GF Seroepidemiological study of respiratory syncytial virus in Sao Paulo State, Brazil J Med Virol 1998 55 234 239 10.1002/(SICI)1096-9071(199807)55:3<234::AID-JMV9>3.0.CO;2-2 9624612

[B25] Ebihara T Endo R Kikuta H Ishiguro N Ishiko H Kobayashi K Comparison of the seroprevalence of human metapneumovirus and human respiratory syncytial virus J Med Virol 2004 72 304 306 10.1002/jmv.10572 14695674

[B26] Pavlin JA Hickey AC Ulbrandt N Chan YP Endy TP Boukhvalova MS Chunsuttiwat S Nisalak A Libraty DH Green S Rothman AL Ennis FA Jarman R Gibbons RV Broder CC Human metapneumovirus reinfection among children in Thailand determined by ELISA using purified soluble fusion protein J Infect Dis 2008 198 836 842 10.1086/591186 18680407 PMC2648801

[B27] Ebihara T Endo R Kikuta H Ishiguro N Ishiko H Hara M Takahashi Y Kobayashi K Human metapneumovirus infection in Japanese children J Clin Microbiol 2004 42 126 132 10.1128/JCM.42.1.126-132.2004 14715742 PMC321731

[B28] Cusi MG Martorelli B Di Genova G Terrosi C Campoccia G Correale P Age related changes in T cell mediated immune response and effector memory to Respiratory Syncytial Virus (RSV) in healthy subjects Immun Ageing 2010 7 14 10.1186/1742-4933-7-14 20961416 PMC2984488

[B29] Nokes DJ Okiro EA Ngama M Ochola R White LJ Scott PD English M Cane PA Medley GF Respiratory syncytial virus infection and disease in infants and young children observed from birth in Kilifi District, Kenya Clin Infect Dis 2008 46 50 57 10.1086/524019 18171213 PMC2358944

[B30] Hall CB Respiratory syncytial virus and parainfluenza virus N Engl J Med 2001 344 1917 1928 10.1056/NEJM200106213442507 11419430

[B31] Marcekova Z Psikal I Kosinova E Benada O Sebo P Bumba L Heterologous expression of full-length capsid protein of porcine circovirus 2 in Escherichia coli and its potential use for detection of antibodies J Virol Methods 2009 162 133 141 10.1016/j.jviromet.2009.07.028 19664658 PMC7119500

[B32] Gao W Soloff AC Lu X Montecalvo A Nguyen DC Matsuoka Y Robbins PD Swayne DE Donis RO Katz JM Barratt-Boyes SM Gambotto A Protection of mice and poultry from lethal H5N1 avian influenza virus through adenovirus based immunization J Virol 2006 80 1959 1964 10.1128/JVI.80.4.1959-1964.2006 16439551 PMC1367171

